# Patologías orales en pacientes pediátricos relacionados con lupus eritematoso sistémico juvenil y consideraciones en el manejo estomatológico. Una revisión

**DOI:** 10.21142/2523-2754-1104-2023-179

**Published:** 2023-12-28

**Authors:** Lizeth Aglaeé Vizcarra Ruiz, Selya Nayjaa Sarmiento Hernández, Juan José Villalobos Rodelo

**Affiliations:** 1 Division de Odontopediatria, Facultad de Odontologia, Universidad Autonoma de Sinaloa. Sinaloa, Mexico. aglaeevizcarra@gmail.com, Universidad Autónoma de Sinaloa Division de Odontopediatria Facultad de Odontologia Universidad Autonoma de Sinaloa. Sinaloa Mexico aglaeevizcarra@gmail.com; 2 Division de Maestria en Odontologia Integral del Nino y Adolescente, Facultad de Odontologia de la Universidad Autonoma de Sinaloa. Sinaloa, Mexico. seylasarmiento@uas.edu.mx, juanvillalobos@uas.edu.mx Universidad Autónoma de Sinaloa Division de Maestria en Odontologia Integral del Nino y Adolescente Facultad de Odontologia Universidad Autonoma de Sinaloa Sinaloa Mexico seylasarmiento@uas.edu.mx juanvillalobos@uas.edu.mx

**Keywords:** lupus eritematoso juvenil, caries dental, manifestaciones orales, odontología pediátrica, juvenile lupus erythematosus, dental caries, oral manifestation, children dentistry

## Abstract

**Introducción:**

El lupus eritematoso juvenil (LESj) es una enfermedad reumática que afecta el funcionamiento de los órganos internos y es multisistémico. Se trata de un padecimiento crónico y suele asociarse con una morbilidad muy significativa, la cual es más elevada en edad infantil y adolescente que en edades adultas.

**Objetivo:**

Describir e identificar los conceptos más actuales del LESj, etiología, epidemiologia de la enfermedad, semiología, manifestaciones orales, así como el tratamiento, consecuencias y diferencias con el lupus eritematoso sistémico en adultos (LESa).

**Materiales y métodos:**

Se realizó búsqueda de la literatura en PubMed, Ebsco, SciELO, Elsevier, utilizando las palabras claves “juvenile lupus erythematosus”, “dental caries”, “oral manifestation”, “children dentistry”. Dirigida a estudios realizados en humanos entre 2010 y 2023, y se analizaron los tópicos más relevantes relacionados a esta enfermedad.

**Resultados:**

La información que se recopiló corresponde a los últimos 13 años, con la finalidad de realizar una actualización sobre el tema de estudio, se revisaron 750 artículos que fueron analizados con los criterios de inclusión y exclusión por lo que solo cumplieron estos criterios 50 artículos.

**Conclusión:**

La atención dental en pacientes con LESj constituye un reto, ya que existen diferentes consideraciones que debemos tomar en cuenta antes de realizar cualquier tratamiento, puesto que presentan alteraciones en articulaciones, glándulas salivales y fallas de múltiples órganos. Es de suma importancia conocer los distintos diagnósticos diferenciales para una inequívoca detección de la enfermedad. Ante la presencia de signos y síntomas basados en los criterios de LESj o de inicio precoz, se recomienda una interconsulta con el área de inmunología para confirmar o descartar esta enfermedad.

## Abstract (en)

**Introduction:**

Juvenile lupus erythematosus (jSLE) is a rheumatic disease that affects the functioning of internal organs and is multisystemic. It is a chronic condition and is usually associated with very significant morbidity, which is higher in children and adolescents than in adults.

**Objectives:**

Describe and identify the most current concepts of jSLE, etiology, epidemiology of the disease, semiology, oral manifestations, as well as treatment, consequences and differences with systemic lupus erythematosus in adults (aSLE).

**Materials and methods:**

A literature search was carried out in PubMed, Ebsco, SciELO, and ELSEVIER, using the key words, “Juvenile lupus erythematosus”,“dental caries”, “oral manifestation”, “children dentistry”. Aimed at studies carried out in humans between 2010 to 2023 and the most relevant topics related to this disease were analyzed.

**Results:**

The information that was collected corresponds to the last 13 years, with the purpose of making an update on the topic of study, 750 articles were reviewed which were analyzed with the inclusion and exclusion criteria but only 50 met these criteria articles.

**Conclusion:**

Dental care in patients with SLEj is a challenge, since there are different considerations that we must take into account before carrying out any treatment, since they present alterations in the joints, salivary glands and failures of multiple organs. It is important to know the different differential diagnoses for unequivocal detection of the disease. In the presence of signs and symptoms based on the criteria of jSLE or early onset, a consultation with the immunology area is recommended to confirm or rule out this disease.

## INTRODUCCIÓN

El lupus eritematoso juvenil (LESj) es una enfermedad reumática que afecta el funcionamiento de los órganos internos y es multisistémico. Se trata de un padecimiento crónico y suele asociarse con una morbilidad muy significativa, la cual es más elevada en edad infantil y adolescente que en edades adultas. Este trastorno se caracteriza principalmente por un descontrol en la producción de anticuerpos, lo que afecta el sistema inmunitario tanto innato como adaptativo, ya que los autoanticuerpos atacan los antígenos nucleares del propio sistema ^1^^-^^4^.

El LESj es una enfermedad inflamatoria y heterogénea, afecta de manera secuencial los sistemas y órganos, con diversas presentaciones clínicas, desde lo que puede ser una sintomatología leve que lentamente puede avanzar hasta posiblemente ser mortal ^3^^,^^5^.

Se denomina inicio precoz de 0 a 9 años; de inicio tardío, de 10 a 19 años. Se ha detectado que tiene una predisposición femenina, aumenta las afectaciones del sistema muscular y esquelético conforme el tiempo avanza ^6^.

Tomasz *et al*. ^7^ destacaron que, con frecuencia, es complicado diferenciar entre los síntomas que todavía se encuentran dentro del desarrollo normal y los que se encuentran considerados como patología. Señala también que, en ocasiones, el LESj se presenta sin algún síntoma que concuerde con sus criterios.

La adolescencia se caracteriza por ser un período en el que existe un aumento de trastornos somáticos y mentales. Esto se puede deber a la alta actividad de los cambios biológicos que ocurren en el individuo, los cuales se pueden tomar como un detonante del LESj ^7^.

Existen síntomas asociados con el área odontológica que deben ser tomados en cuenta como las úlceras orales, los trastornos de las articulaciones en los que se ve afectada la articulación temporomandibular, deficiencias en la producción de saliva, además de considerar el aspecto psicológico por el que está atravesando el paciente con LESj. El presente estudio tiene como propósito principal aportar una herramienta al odontólogo que le permita brindar un tratamiento adecuado a este tipo de pacientes, así como la capacitación en la detección oportuna de los casos de LESj no diagnosticados para la posterior remisión a las respectivas áreas de salud.

## MATERIAL Y MÉTODOS

Se utilizaron bases de datos como PubMed, Ebsco, SciELO, Elsevier, con las palabras claves utilizadas como herramienta de búsqueda fueron “juvenile lupus erythematosus”, “dental caries”, “oral manifestation” y “children dentistry”.

Los criterios de selección utilizados para determinar la búsqueda fueron:

### Criterios de inclusión:

Artículos originales, casos clínicos, diseños de cohortes, metaanálisis, artículos en inglés y español, estudios realizados en humanos, publicados entre el 2010 y 2023, que incluyan individuos con rango de edad de 2 hasta 50 años, en los que se pudieron realizar comparaciones de las características en los inicios de la enfermedad (LESj y LESa)

### Criterios de exclusión:

Artículos con más 13 años de publicación, estudios en pacientes con lupus eritematoso sistémico que presentaban otra enfermedad no secundaria a lupus, y casos clínicos en los cuales los pacientes no finalizaron el estudio

## RESULTADOS

El proceso de búsqueda en las bases de datos virtual incluyó un total de 750 artículos, de los cuales solo 50 cumplieron los criterios de selección.

### Causas del lupus eritematoso sistémico

Entre las causas del LESj se destacan factores hereditarios, problemas durante la gestación y etapa neonatal. También pueden favorecer este trastorno problemas hormonales y exposiciones ambientales como largos periodos de exposición solar, infecciones virales y contaminación atmosférica ^5^.

El LESj está fuertemente relacionado a las hormonas; por este hecho, 9 de cada 10 personas que lo padecen suelen ser mujeres. Además, la enfermedad aparece con frecuencia en edades reproductivas. En el LES de inicio temprano no se encontró una correlación significativa tan importante como en los de inicio juvenil o adulto. Por otra parte, la aparición en LES de inicio precoz puede tener mayor asociación con la carga genética ^8^.

Las características que conducen a la aparición de LESj son complejas y suelen ser diferentes entre cada paciente, ya que influyen factores como el sexo y la edad (las manifestaciones clínicas son más agresivas en edades tempranas) ^9^^,^^10^.

El 1% de la población desarrolla la enfermedad como resultado de mutaciones genéticas y el porcentaje restante son portadores de variantes genéticas. En los pacientes con LESj, la carga genética tiene mayor predisposición que en el LES en edad adulta. 

Este padecimiento presenta una mayor actividad de las afectaciones causadas por la enfermedad, lo que requiere de tratamientos más agresivos. Se considera que entre un 10% y un 20% de los pacientes que padecen LES la enfermedad se desarrolló antes de cumplir los 16 años ^11^^-^^14^. 

Hendrich ^13^ señala factores adicionales que pueden influenciar la aparición del LESj, entre ellos problemas hormonales, toxinas, infecciones recurrentes, altas exposiciones a radiaciones UV y alteraciones epigenéticas, además de enfermedades secundarias no tan conocidas como microangiopatías trombóticas secundarias a LESj ^14^^-^^16^. 

La patogenia del LESj está mediada por la formación de complejos inmunitarios solubles que con frecuencia involucran autoanticuerpos contra proteínas nucleares, observándose autoanticuerpos en contra del ADN de doble cadena. Estos conducen a una sobreproducción de interferones tipo I y otras citosinas, al igual que la participación de la inmunidad mediada por células e implicación humoral consecuente. El complejo inmune depositado en diferentes órganos desencadena un proceso inflamatorio, esta reacción produce deterioro y daño tisular ^3^^,^^17^^,^^18^. 

### Epidemiologia

El LESj es una enfermedad que se puede encontrar en todo el mundo con una predilección por las mujeres en edades fértiles de entre 14 y 55 años. Si bien está presente en todas las etnias es encontrada con mayor frecuencia en personas no caucásicas ^19^.

Cosier *et al*. ^20^ señalan que la exposición a situaciones de estrés puede ayudar a la aparición del LES a través del desbalance de las respuestas adaptativas al estrés frecuente. Un estudio reciente resalta una evaluación hecha a mujeres de raza negra que sufrieron abuso físico y sexual en la infancia; estos factores se asociaron con el aumento estadísticamente significativo en el diagnóstico de LES.

### Prevalencia e incidencia

Se tomaron en cuenta 6 artículos para la revisión de incidencia, prevalencia, sexo y predominio racial de la enfermedad. Resalta un predominio en la raza afrodescendiente y el sexo femenino en el LESj, datos que se encuentran reflejados en la Tabla 1 ^21^^-^^26^.

**Tabla 1:** Distribución de incidencia y prevalencia en distintas poblaciones

N.°	Incidencia	Prevalencia	Predominio	Tendencia Racial.	Año	Bibliografía
1	4,9 por C/100,000	0,72 por C/100,000	Femenino	Reino Unido	2019	Tomado de Varnier ^1^
2	0,3 a 0,9 por C/100,000	3,3 a 8,8 C/100,000	Femenino	Asiáticos, afroamericanos, hispanos y nativos americanos	2012	Tomado de Levy DM ^22^
3	0,3 a 0,9 por C/100,000	1,89 y 25,7 por C/100,000	Femenino	Sin predominio tacial.	2017	Tomado de Groot ^23^
4	0,28 y 0,48 por C/ 100,000	3,3 y 24,0 por C/ 100,00	Femenino	Afroamericanos, hispanos, blancos y residentes del sur de EE. UU.	2012	Tomado de Hiraki ^24^
5	0,36 y 2,5 por C/100,000	1,89 y 34,1 por C/100,000	Femenino Reino Unido, Turquía, Egipto, Portugal y EE. UU	.	2019	Tomado de Smith ^9^
6	0,28 a 0,9 por C/ 100,000	500 casos por C/ millón	Femenino	Varias poblaciones raciales, étnicas y población asiática	2014	Tomado de Tavangar ^25^

### Criterios de clasificación

Existen diferentes criterios de clasificación y los más usuales son los siguientes: 

1. ACR (American College of Rheumatology), 1997

2. SLICC (Systemis Lupus International Collaborating Centers), 2021

3. EULAR (Liga Europea de Reumatismo), 2019 ^12^

Según los nuevos criterios del SLICC, la presencia de nefropatías, más un criterio inmunológico como el ANA o el ADNds, serían suficientes para el diagnóstico del paciente ^26^.

### Métodos de diagnóstico

Se utilizan diferentes métodos para el diagnóstico, tanto clínicos como estudios de laboratorio, en los cuales se debe tomar en cuenta por lo menos un criterio clínico y otro inmunológico ^27^.

Dentro de los estudios de laboratorio para determinar el diagnóstico se encuentra el de anticuerpos antinucleares (ANA) y anticuerpos antipéptidos cíclico (Anti Ds DNA), Anti-RO y Anti-La.

Los ANA son los primeros en estar en el suero. Estos se unen a varios antígenos nucleares y citoplasmáticos, y son descritos como un biomarcador sensible ante la evaluación de sospecho del inicio de enfermedades reumáticas; sin embargo, no son útiles para monitorear la actividad de la enfermedad. Entre los exámenes primarios para la prueba de ANA se pueden destacar 3 tipos: inmunoensayos enzimáticos, inmunoensayos multiplex e inmunofluorescencia indirecta en ensayo en células, siendo este último el estándar de oro ^18^.

Una vez detectado el LESj se realizan estudios en los que se busca identificar diferentes afectaciones como trastornos renales, neurológicos, hematológicos, artritis, problemas hepáticos, pulmonares, etc. ^22^^,^^28^.

### Manifestaciones generales

Podemos destacar, entre los signos y síntomas clínicos, los siguientes:

• Erupción malar: se caracteriza por un eritema plano o elevado localizado en la eminencia malar, que no incluye los pliegues nasolabiales.
• Erupción discoide eritematosa.
• Fotosensibilidad: la cual se presenta con erupciones en la piel por la exposición solar.
• Úlceras orales, nasofaríngeas: mayormente detectadas en el paladar blando y duro.
• Artritis no erosiva: puede involucrar 2 o más articulaciones, las cuales suelen ser periféricas y se caracterizan por producir sensibilidad e hinchazón.
• Serositis pleuritis o pericarditis: es un dolor localizado, agudo y fugaz.
• Trastornos renales.
• Trastornos neurológicos: mayormente detectados en convulsiones.
• Trastornos hematológicos, de las cuales destacan la anemia hemolítica, la leucopenia, la linfopenia y la trombocitopenia.
• Trastornos inmunológicos y anticuerpos anti nucleares positivos.
• Bajo c3 ^(1, 9, 22)^.

En la Tabla 2 se enumeran las manifestaciones más comunes en pacientes con LESj y LESa descritas por Livingston *et al*. ^10^ y Tarr *et al*. ^29^.

**Tabla 2:** Manifestaciones más comunes en lupus eritematoso sistémico juvenil y lupus eritematoso sistémico adulto

LESj	LESa
Fiebre	Síndrome del Goseco
Linfadenopatia	Raynaud
Erupción molar	Pleuresía
Úlceras mucocutáneas	Afectaciones renales
Proteinuria	Erupción molar
Cilindros celulares	Poliartritis
Afectaciones renales generales	S. neurológicos
Convulsiones	Serositis
Tromboatopenia	Fotosensibilidad
Anemia hemolítica	

Bibliografía: Tomado de Livingston ^10^ y Tarr ^25^

El lupus eritematoso sistémico se presenta con una serie de síntomas que constituyen fiebre, fatiga, alopecia, inflamaciones, posible pérdida de peso, afectaciones en el SNC y músculoesquelético, así como mayor predisposición a padecimientos renales ^21^^,^^30^.

Fariba Tavangar-Rad *et al*. ^27^ realizaron un estudio en 120 pacientes pediátricos, 92 niñas y 28 varones, para determinar la frecuencia de morbilidad. Se destacan las afectaciones mostradas en el grafico 1: afectaciones articulares, mucocutáneas, hematológicas y endocrinas.

El diagnóstico precoz del LES es crucial para prevenir los brotes y el daño tisular. Es importante considerar que el camino hacia el LES comienza antes de las características clínicas ^18^.

### Tratamientos

Existe una amplia variedad de medicamentos para el control del LESj, los cuales varían dependiendo de la gravedad de la enfermedad, si son indicados para terapias de inducción o terapias de mantenimiento. Podemos destacar de esta serie de fármacos los efectos secundarios que tienen en el paciente, como se aprecia en la Tabla 3 ^31^.

**Tabla 3:** Medicamentos y sus efectos secundarios usados en el control de lupus eritematoso sistémico juvenil

N.°	Medicación	Indicaciones	Efectos Secundarios
1	Glucocorticoesteroides	Terapia de inducción y mantenimiento leves o no remitente	- Supresión suprarrenal
			- Estrías
			- Obesidad
			- Cambios de humor
			- Fracaso del crecimiento
			- Osteoporosis
2	Ciclofosfamida	Terapia de inducción y intravenosa moderada a grave con afectación a órganos	- Infertilidad
			- Caída de cabello
			- Mayor riesgo de infección
			-Náuseas y vómito
			- Riesgo de malignidad a largo plazo
3	Micofenolato mofetilo	Terapia de inducción y mantenimiento de enfermedad moderada a grave	- Molestias abdominales
			- Diarrea
			- Inflamación hepática
			- Mayor riesgo de infección
			- Teratogénico en el embarazo
4	Azatioprina	Terapia de mantenimiento en enfermedad leve, moderada o grave	- Supresión de médula ósea
			- Mayor riesgo de infección
5	Metotrexato	Terapia de mantenimiento en síntomas musculo-esquelético	- Supresión de la médula ósea
			- Náuseas y vómito
			- Inflamación hepática
6	Hidroxicloroquina	Todos los PX	- Evitar en embarazos o deficiencia de G6PD

Bibliografía: Tomado de Thorbison ^31^

Dentro de los tratamientos generales se recomienda a los pacientes con LESj el uso de protectores solares a todas horas del día, una dieta equilibrada y baja en sodio, el consumo de alimentos y suplementos con calcio e inmunosupresores. Entre los más frecuentes encontramos los siguientes:

• Prednisona
• Micofenolato mofetilo
• Azatioprina
• Hidroxicoloquina
• Ciclofosfamida
• Rituximab ^(5, 6)^

### Manifestaciones odontológicas

Es importante para el odontólogo detectar, con base en los signos y síntomas, alteraciones o enfermedades que presente el paciente y llevar a cabo un tratamiento eficaz y conservador. De la misma manera, es conveniente que se encuentre preparado para englobar los síntomas de sospecha de LESj, aun cuando no esté diagnosticado por el área de reumatología. Las manifestaciones orales son comunes en el LESj y en el LESa en las primeras etapas de la aparición de la enfermedad. Las afectaciones dermatológicas suelen aparecer después de que existe una falla del sistema hematológico, muscular y esquelético ^32^.

Direkrit Chiew *et al*. ^32^ determinaron que la aparición de alteraciones mucocutáneas era más frecuente en el LESj. Asimismo, Rodsaward *et al*. ^33^ observaron que las úlceras orales son la segunda alteración más frecuente, por debajo de la erupción mariposa, mientras que Khatibi *et al*. ^34^ llegaron a la conclusión de que no existe relación aparente en las alteraciones orales con los fármacos consumidos en el tratamiento del control del LESj. 

Nazri *et al*. ^35^ realizó un estudio retrospectivo con 32 pacientes en el Hospital Universiti Sains Malaysia (Kelantan, Malasia), en la cual las úlceras orales aparecieron en un 21,9% de los casos.

Dentro de las alteraciones de la mucosa oral se observan algunas no tan comunes en LESj, pero que es posible detectar ^36^. Entre ellas se encuentran la siguientes:

• Erosión de la mucosa
• Placas de superficie descamativa
• Fisuras con tendencia hemorrágica
• Ulceraciones y alteración dentro de las placas
• Estrías blancas o lesiones discoides
• Máculas eritematosas extensas

Se pueden tener diagnósticos diferenciales de las úlceras orales, tales como lesiones aftosas, lesiones vesiculares ampollosas y enfermedad de manos, pies y boca, por lo que es necesario considerar tanto la historia clínica odontológica como la historia clínica general del paciente ^37^.

La mayor parte de las enfermedades autoinmunes afectan la piel y la mucosa; esta última incluye la cavidad oral. Las principales lesiones que involucran la cavidad oral de manera ampollosa son las siguientes:

• Pénfigo ampolloso
• Penfigoide mucoso
• Penfigoide ampolloso
• Liquen plano oral ^38^

Las lesiones penfigoides, son enfermedades ampollosas subdérmica formadas por erupciones cutáneas polimórficas con sangre y ampollas llenas de líquido. Se caracteriza por autoanticuerpos que afectan las estructuras que componen la unión dermoepidérmica ^39^.

La dificultad para el odontólogo radica en que estas alteraciones tienen aspectos clínicos muy similares, por lo que únicamente se determina su diagnóstico con una evaluación histológica y apoyo multidisciplinario. Es común que estas alteraciones se presenten en los inicios de la enfermedad; de esta forma, el odontólogo juega un papel clave en el diagnóstico inicial ^36^^,^^38^.

Además de las alteraciones de la mucosa oral, encontramos que la condición periodontal es peor en pacientes con LES Y LESj. Está asociada con altos niveles de interleucina (1L)-1B-1L8, estimulación de factores de colonias de granulocitos (G-CSF), interferón y proteína quimioatrayente de monocitos en el líquido cervical gingival. Esto se determinó en un estudio de Sete *et al*. ^40^, el cual consistió en examinar a 21 pacientes con LESj. Se investigaron datos reumatológicos y periodontales, y todos los pacientes fueros diagnosticados con inflamación gingival. El tratamiento consistió en raspado supragingival, profilaxis e instrucciones de higiene oral. Se recabó información del inicio a 6 meses de seguimiento revisando los niveles de citosina y los niveles de Anti-PPAD mediante ELISA, y se recogieron muestras de fluido cervicular gingival. Se concluyó que los pacientes con LESj se beneficiaron del tratamiento periodontal, tuvieron una reducción de LCG (líquido cervical gingival) de citocinas proinflamatorias y un aumento de las antinflamatorias. 

Sete *et al*. ^41^ realizaron un estudio en el cual evaluaron los niveles de varias citocinas en el LCG y el suero en pacientes con LESj e inflamación gingival. Recabaron información de 29 pacientes sanos y 30 con LESj. Los participantes fueron examinados reumatológica y periodontalmente; se recolectó LCG, suero y placa dentobacteriana, las citosinas se analizaron mediante ensayos multiplex basados en perlas y el perfil bacteriano, mediante hibridación de ADN. Los pacientes con LESj presentaron mayor porcentaje de placa dentobacteriana y sangrado que los controles, una profundidad media al sondaje y pérdida de inserción. En conclusión, se comprobó que los pacientes con LESj presentaron una condición periodontal inferior asociada con niveles alterados de citocinas proinflamatorias en LCG y un mayor recuento de *A. Actinomycetemcomitans* en el biofilm dental.

Van der Meulen *et al*. ^42^ destacaron que los pacientes con LESj tienen una menor riqueza bacteriana y, a su vez, una mayor predisposición a padecer síndrome de Sjögren como efecto secundario. 

Katz *et al*. ^43^ realizaron un reporte de caso en el que una paciente de 15 años 9 meses y origen hispano llegó al departamento de odontopediatría refiriendo presentar una úlcera en el paladar con una duración de 6 meses. El examen extraoral mostró que presentaba lo siguiente:

• Linfadenopatías cervical, cervical posterior y submandibular
• Erupción malar hiperpigmentada
• Agrandamiento de la glándula parótida derecha y lóbulo inferior palpable
• Moderación en la cantidad salival
• Múltiples lesiones sobrelevadas en cara ventral de la lengua
• Lesiones exofíticas de 1,5 cm en paladar blando y duro
• Numerosas úlceras en encía

En la historia clínica general, se identificó lo siguiente:

• Cefaleas
• Fatiga en los últimos 3 años
• Poliatralgias (manos, rodillas y tobillos)
• Rigidez matutina
• Fiebre de 37,5 °C a 38 °C
• Sudores nocturnos ^24^

En la Tabla 4 se aprecian las alteraciones odontológicas del LESj.

**Tabla 4:** Principales manifestaciones orales en pacientes con LESj

Manifestaciones orales	Bibliografía
- Labio inflamado	Tomado de Schildt *et al*. ^44^
- Ulceración y sangrado	
- Odinofagia	
- Cansancio	
-Fiebre	
-Pérdida de peso	
- Angioedemas	
- Queilitis	
- Overbite disminuido	Tomado de Golin *et al*. ^45^
- Overjet aumentado	
- Mordida cruzada	
- Clase 1	
- Cambio de línea media dental	
- Apiñamiento sup.	
- Disgeusia	Tomado de Crincoli *et al*. ^46^
- Estomatodinia	
-Dolor en músculos masticatorias	
- Artralgia	
- Petequias	
- Eritema	
- Lengua fisurada	
- Queilitis de labios inferior	
- Hiposalivación	
- Bruxismo	
- Trastornos de apertura.	
- Úlceras orales	Tomada de García-Ríos *et al*. ^47^ y Müller *et al*. ^48^
- Pigmentación oral	
- Hiperqueratosis	
- Erosión	
- Flujo salival alterado	
- Artritis	

### Consideraciones odontológicas en pacientes con LESJ

Se deben tomar en cuenta las alteraciones secundarias a LESj en los tratamientos dentales, ya que pueden dificultar la atención al paciente. Se deben idear estrategias que permitan modificar los tratamientos para cada paciente y tener un menor tiempo operatorio.

Lo recomendable es trabajar con interconsultas de la mano del departamento de inmunología, erradicar los focos de infección y llevar a cabo un seguimiento para mejorar la salud oral del paciente. También es importante considerar que algunos fármacos utilizados (antinflamatorios esteroideos e inmunosupresores) afectan a nivel hematológico, por lo que estos pacientes pueden modificar la producción de células de defensa y reparación tisular, de manera que resulta importante iniciar con profilaxis antibiótica antes del tratamiento dental ^44^^-^^49^.

Pascali *et al*. ^50^ destacan en su artículo las consideraciones psicológicas que pueden alterar las emociones y desestabilizar las conductas de los niños. Esto se deriva del dolor crónico producido por la misma enfermedad (LESj), así que se debe ser empático al realizar una intervención operatoria dental en pacientes con LESj. 

Tomasz *et al*. ^7^ reportan el caso de una paciente de 16 años cuyo único síntoma era depresión severa y quien no tuvo síntomas somáticos durante un largo periodo de tiempo. 

Skakodub *et al*. ^51^ proponen una llave de silicón hecha a medida, lo que resulta muy útil ante la apertura limitada. La paciente también presentaba esclerosis de los músculos circulares de la boca y formación de microstomías. Utilizaron también composites en lesiones cariosas, ya que la inflamación es un problema importante en la dentición.

Es importante resaltar que el tratamiento dental se debe posponer ante la presencia de autoanticuerpos activos, anemia, leucopenia, neutropenia o trombocitopenia. En algunos de los casos se pueden efectuar modificaciones en la toma de los anticoagulantes o auxiliarse con hemostáticos locales, ya que es importante eliminar cualquier foco de infección en la cavidad oral del paciente ^49^.

## CONCLUSIONES

El LESj es un trastorno inmunológico más conocido en la actualidad, con severas consecuencias a nivel multisistémico, las cuales son aún más graves y evolucionan con mayor rapidez en edades tempranas.

El involucramiento del profesional en odontología es de suma importancia para la detección temprana del LESj, ya que las manifestaciones mucocutáneas son las primeras en aparecer. Se debe tener en cuenta que existen consideraciones clínicas destacadas antes de realizar algún tratamiento, entre las que destacan alteraciones en articulaciones, glándulas salivales y fallas de múltiples órganos, por lo que es imperativo conocer los distintos diagnósticos diferenciales para una inequívoca detección de la enfermedad. Ante la presencia de signos y síntomas basados en los criterios de LESj o de inicio precoz, se recomienda la interconsulta con el área de inmunología para detectar o descartar esta enfermedad. 

También es importante recalcar y considerar los cambios psicológicos que presentan estos pacientes, ya que pueden modificar la conducta del niño durante las consultas. Se debe recordar que cada caso debe ser individualizado a las necesidades requeridas y condición de salud de cada paciente.

## Fuente de financiamiento:

Autofinanciado

## References

[1] Varnier GC, Ciurtin C (2019). Paediatric and adolescent rheumatic diseases measures of disease activity. Br J Hosp Med (Lond).

[2] Watson L, Leone V, Pilkington C, Tullus K, Rangaraj S, McDonagh JE (2012). Disease activity, severity, and damage in the UK Juvenile-Onset Systemic Lupus Erythematosus Cohort. Arthritis Rheum.

[3] Hamburger J (2016). Orofacial manifestations in patients with inflammatory rheumatic diseases. Best Pract Res Clin Rheumatol.

[4] Cavallaro M, Barbaro U, Caragliano A, Longo M, Cicero G, Granata F, Racchiusa S (2018). Stroke and systemic lupus erythematosus: a review. Rheumatology.

[5] Trindade VC, Carneiro-Sampaio M, Bonfa E, Silva CA (2021). An Update on the Management of Childhood-Onset Systemic Lupus Erythematosus. Paediatr Drugs.

[6] Nelson MC, Chandrakasan S, Ponder L, Sanz I, Goldberg B, Ogbu EA, Rouster-Stevens K, Prahalad S (2022). Clinical Determinants of Childhood Onset Systemic Lupus Erythematosus among Early and Peri-Adolescent Age Groups. Children (Basel).

[7] Koszutski T, Wiernik A, Hyla-Klekot L, Matysiak N, Kucharska G (2022). The role of assessment of renal biopsy in electron microscopy in making a diagnosis of juvenile-onset systemic lupus erythematosus in a 16-year-old female patient with depression and proteinuria - a case report. Cent Eur J Immunol.

[8] Olmos-García FX, Suárez-Larios LM, Velázquez-Contreras CA, Sotelo-Cruz N, Manjarrez-Orduño N (2014). Lupus Eritematoso Sistémico en la Edad Pediátrica. Boletín Clínico Hospital Infantil del Estado de Sonora.

[9] Smith EMD, Lythgoe H, Midgley A, Beresford MW, Hedrich CM (2019). Juvenile-onset systemic lupus erythematosus Update on clinical presentation, pathophysiology and treatment options. Clin Immunol.

[10] Livingston B, Bonner A, Pope J (2011). Differences in clinical manifestations between childhood-onset lupus and adult-onset lupus a meta-analysis. Lupus.

[11] Charras A, Smith E, Hedrich CM (2021). Systemic Lupus Erythematosus in Children and Young People. Curr Rheumatol Rep.

[12] Ameer MA, Chaudhry H, Mushtaq J, Khan OS, Babar M, Hashim T, Zeb S, Tariq MA, Patlolla SR, Ali J, Hashim SN, Hashim S (2022). An Overview of Systemic Lupus Erythematosus (SLE) Pathogenesis, Classification, and Management. Cureus.

[13] Hedrich CM, Smith EMD, Beresford MW (2017). Juvenile-onset systemic lupus erythematosus (jSLE) - Pathophysiological concepts and treatment options. Best Pract Res Clin Rheumatol.

[14] Dung NT, Loan HT, Nielsen S, Zak M, Petersen FK (2012). Juvenile systemic lupus erythematosus onset patterns in Vietnamese children a descriptive study of 45 children. Pediatr Rheumatol Online J.

[15] Kaneda T, Tanaka E, Akutsu Y, Kanamori T, Mouri M, Morio T, Mori M (2020). Refractory secondary thrombotic microangiopathy with kidney injury associated with systemic lupus erythematosus in a pediatric patient. CEN Case Rep.

[16] Zucchi D, Silvagni E, Elefante E, Signorini V, Cardelli C, Trentin F, Schilirò D, Cascarano G, Valevich A, Bortoluzzi A, Tani C (2023). Systemic lupus erythematosus one year in review 2023. Clin Exp Rheumatol.

[17] Saccucci M, Di Carlo G, Bossù M, Giovarruscio F, Salucci A, Polimeni A (2018). Autoimmune Diseases and Their Manifestations on Oral Cavity Diagnosis and Clinical Management. J Immunol Res.

[18] Lazar S, Kahlenberg JM (2023). Systemic Lupus Erythematosus New Diagnostic and Therapeutic Approaches. Annu Rev Med.

[19] Fava A, Petri M (2019). Systemic lupus erythematosus Diagnosis and clinical management. J Autoimmun.

[20] Cozier YC, Barbhaiya M, Castro-Webb N, Conte C, Tedeschi S, Leatherwood C, Costenbader KH, Rosenberg L (2021). Association of Child Abuse and Systemic Lupus Erythematosus in Black Women During Adulthood. Arthritis Care Res (Hoboken).

[21] Jiménez-Uscanga RD, Carsolio-Trujano M, Herrera-Sánchez DA, Castrejón-Vázquez MI, Irazoque-Palacios F, Vargas-Camaño ME, Martínez-Aguilar N, Chima-Galán MC (2015). Lupus eritematoso sistémico y CD24v (Systemic lupus erythematous and CD24v). Rev Alerg Mex.

[22] Levy DM, Kamphuis S (2012). Systemic lupus erythematosus in children and adolescents. Pediatr Clin North Am.

[23] Groot N, de Graeff N, Avcin T, Bader-Meunier B, Brogan P, Dolezalova P, Feldman B, Kone-Paut I, Lahdenne P, Marks SD, McCann L, Ozen S, Pilkington C, Ravelli A, Royen-Kerkhof AV, Uziel Y, Vastert B, Wulffraat N, Kamphuis S, Beresford MW (2017). European evidence-based recommendations for diagnosis and treatment of childhood-onset systemic lupus erythematosus the SHARE initiative. Ann Rheum Dis.

[24] Hiraki LT, Feldman CH, Liu J, Alarcón GS, Fischer MA, Winkelmayer WC, Costenbader KH (2012). Prevalence, incidence, and demographics of systemic lupus erythematosus and lupus nephritis from 2000 to 2004 among children in the US Medicaid beneficiary population. Arthritis Rheum.

[25] Tavangar-Rad F, Ziaee V, Moradinejad MH, Tahghighi F (2014). Morbidity and Mortality in Iranian Children with Juvenile Systemic Lupus erythematosus. Iran J Pediatr.

[26] Caggiani M, Gazzara G (2003). Lupus eritematoso sistémico en niños y adolescentes: características clínicas, inmunológicas y evolutivas. Análisis y consideraciones terapéuticas. Arch Pediatr Urug.

[27] Ranjan R, Mehta S, Saxena KN (2021). Unveiling Uncommon Manifestations in a Pediatric Patient With Systemic Lupus Erythematosus A Case Report. Cureus.

[28] Groot N, de Graeff N, Avcin T, Bader-Meunier B, Brogan P, Dolezalova P (2017). European evidence-based recommendations for diagnosis and treatment of childhood-onset systemic lupus erythematosus the SHARE initiative. Ann Rheum Dis.

[29] Tarr T, Dérfalvi B, Gyori N, Szántó A, Siminszky Z, Malik A, Szabó AJ, Szegedi G, Zeher M (2015). Similarities and differences between pediatric and adult patients with systemic lupus erythematosus. Lupus.

[30] Herrera CN, Bohórquez Velasco M, Tomalá Haz J (2020). Lupus eritematoso sistémico juvenil: características clínicas e inmunológicas en una cohorte ecuatoriana de 71 pacientes, experiencia de un Centro. Rev Med UCSG.

[31] Thorbinson C, Oni L, Smith E, Midgley A, Beresford MW (2016). Pharmacological Management of Childhood-Onset Systemic Lupus Erythematosus. Paediatr Drugs.

[32] Chiewchengchol D, Murphy R, Edwards SW, Beresford MW (2015). Mucocutaneous manifestations in juvenile-onset systemic lupus erythematosus a review of literature. Pediatr Rheumatol Online J.

[33] Rodsaward P, Prueksrisakul T, Deekajorndech T, Edwards SW, Beresford MW, Chiewchengchol D (2017). Oral Ulcers in Juvenile-Onset Systemic Lupus Erythematosus A Review of the Literature. Am J Clin Dermatol.

[34] Khatibi M, Shakoorpour AH, Jahromi ZM, Ahmadzadeh A (2012). The prevalence of oral mucosal lesions and related factors in 188 patients with systemic lupus erythematosus. Lupus.

[35] Nazri SKSM, Wong KK, Hamid WZWA (2018). Pediatric systemic lupus erythematosus Retrospective analysis of clinico-laboratory parameters and their association with Systemic Lupus Erythematosus Disease Activity Index score. Saudi Med J.

[36] Alemán MO, Morales ND, Jardón CJ (2020). Evolución del estudio de las manifestaciones bucomaxilofaciales del lupus eritematoso sistémico. Rev Cub de Reu.

[37] Horri A, Danesh M, Sadat Hashemipour M (2020). Childhood Systemic Lupus Erythematosus; a Rare Multisystem Disorder Case Report of a 3-year-old Girl with Oral Involvement as a Primary Sign. J Dent (Shiraz).

[38] Mays JW, Sarmadi M, Moutsopoulos NM (2012). Oral manifestations of systemic autoimmune and inflammatory diseases diagnosis and clinical management. J Evid Based Dent Pract.

[39] Mustafa MB, Porter SR, Smoller BR, Sitaru C (2015). Oral mucosal manifestations of autoimmune skin diseases. Autoimmun Rev.

[40] Sete MRC, Carlos JC, Mello-Neto JM, Lira-Junior R, Brito F, Bostrom EA, Sztajnbok FR, Figueredo CM (2021). Impact of chronic gingivitis management on the cytokine and anti-PPAD expressions in juvenile systemic lupus erythematosus A six-month follow-up. J Periodontal Res.

[41] Sete MRC, Carlos JC, Lira-Junior R, Bostrom EA, Sztajnbok FR, Figueredo CM (2019). Clinical, immunological and microbial gingival profile of juvenile systemic lupus erythematosus patients. Lupus.

[42] Van der Meulen TA, Harmsen HJM, Vila AV, Kurilshikov A, Liefers SC, Zhernakova A (2019). Shared gut, but distinct oral microbiota composition in primary Sjogren&apos;s syndrome and systemic lupus erythematosus. J Autoimmun.

[43] Katz J, Islam MN, Cha S, Saleh W, Guelmann M (2020). Persistent Palatal Ulcerations A Potential Manifestation of Juvenile Systemic Lupus Erythematosus. J Clin Pediatr Dent.

[44] Schildt E, Sessions KL, De Ranieri D (2021). Juvenile Systemic Lupus Erythematosus Presenting with Esophagitis and Severe Oral Mucositis. Case Rep Rheumatol.

[45] Golin SL, Sinicato NA, Valle-Corotti K, Fuziy A, Nahas-Scocate AC, Appenzeller S, Costa ALF (2017). Assessment of condyle, masseter and temporal muscles volumes in patients with juvenile systemic lupus erythematosus A cross-sectional study. J Oral Biol Craniofac Res.

[46] Crincoli V, Piancino MG, Iannone F, Errede M, Di Comite M (2020). Temporomandibular Disorders and Oral Features in Systemic Lupus Erythematosus Patients An Observational Study of Symptoms and Signs. Int J Med Sci.

[47] García-Ríos P, Pecci-Lloret MP, Oñate-Sánchez RE (2022). Oral Manifestations of Systemic Lupus Erythematosus A Systematic Review. Int J Environ Res Public Health.

[48] Muller L, van Waes H, Langerweger C, Molinari L, Saurenmann RK (2013). Maximal mouth opening capacity percentiles for healthy children 4-17 years of age. Pediatr Rheumatol Online J.

[49] Gómez-Contreras P, De la Teja-Ángeles E, Ceballos-Hernández H, Elías-Madrigal G, Estrada-Hernández E, Gutiérrez-Hernández A (2015). Tratamiento estomatológico interdisciplinario del lupus eritematoso generalizado. Presentación de un caso. APM.

[50] Pascali M, Matera E, Craig F, Torre F, Giordano P, Margari F, Zagaria G, Margari M, Margari L (2019). Cognitive, emotional, and behavioral profile in children and adolescents with chronic pain associated with rheumatic diseases A case-control study. Clin Child Psychol Psychiatry.

[51] Skakodub AA, Mamedov AA, Admakin OI, Dudnik OV, Chertikhina AS, Beznosik AR (2022). Treatment of Fissure Caries of Children with Severe Rheumatic Diseases with Difficulty in Opening the Mouth. Contemp Clin Dent.

